# Gold Nanoparticles for Qualitative Detection of Deltamethrin and Carbofuran Residues in Soil by Surface Enhanced Raman Scattering (SERS)

**DOI:** 10.3390/ijms20071731

**Published:** 2019-04-08

**Authors:** Yong He, Shupei Xiao, Tao Dong, Pengcheng Nie

**Affiliations:** 1 College of Biosystems Engineering and Food Science, Zhejiang University, Hangzhou 310058, China; yhe@zju.edu.cn (Y.H.); 180312@zju.edu.cn (S.X.); 21613052@zju.edu.cn (T.D.); 2 Key Laboratory of Spectroscopy Sensing, Ministry of Agriculture and Rural Affairs, Hangzhou 310058, China; 3 State Key Laboratory of Modern Optical Instrumentation, Zhejiang University, Hangzhou 310058, China

**Keywords:** deltamethrin, carbofuran, pesticide residues in soil, surface-enhanced Raman spectroscopy, gold nanoparticles

## Abstract

The residues of deltamethrin (DM) and carbofuran (CBF) in soil is becoming an intractable problem causing soil hardening and environmental pollution. This paper reports a very simple method via improved reduction of chloroauric acid by the trisodium citrate method for fabricating gold nanoparticles (AuNPs), which were used as a surface enhanced Raman scattering (SERS) active colloids with the advantages of ultrasensitivity, reproducibility and chemical stability. The results demonstrated that the limits of detection (LODs) of the DM and CBF were found to be as low as 0.01 mg/L. The SERS intensity showed a good linear relationship with DM (*R*^2^ = 0.9908) and CBF (*R*^2^ = 0.9801) concentration from 0.01 to 10 mg/L. In a practical application, DM and CBF residues in soil were easily detected by SERS with the flexible AuNPs colloids, and the LODs of DM and CBF were found to be as low as 0.056 mg/kg and 0.053 mg/kg, respectively. Moreover, DM in soil could be qualitatively detected by the characteristic peaks at 560 and 1000 cm^−1^, and CBF in soil could be qualitatively detected by the characteristic peaks at 1000 and 1299 cm^−1^. The determination coefficient (R^2^_p_) for DM and CBF reached 0.9176 and 0.8517 in partial least squares (PLS) model. Overall, it is believed that the prepared AuNPs can provide technical support for the accurate detection of pesticide residues in soil by SERS technique.

## 1. Introduction

Pesticides are indispensable means of production in modern agriculture, which have been used to control a large variety of insects, fungi, viruses, and weeds [1,2,3]. As highly effective and toxic insecticides, deltamethrin (DM) and carbofuran (CBF) have been used worldwide in agriculture for the better control of crop pests. However, soil pollution is becoming more and more serious due to the long-term abuse and overuse of pesticides. The general pollution exposure to the pesticides destroys soil biodiversity, endangers plant growth, and threatens human health [4,5]. Therefore, the rapid diagnosis of pesticide residues in soil is essential for soil remediation and bio-environment safety. Currently, the traditional techniques for determining pesticides in soil include gas chromatography (GC), high performance liquid chromatography (HPLC) [6], gas chromatography–mass spectrometry (GC–MS) and liquid chromatography–mass spectrometry (LC–MS) [7]. And the detected limits of detection (LODs) of pesticides in soil are generally at the trace level (mg/kg) or ultra-trace level (μg/kg). Ali, et al. applied HPLC to determine the triazine pesticides (ametryn, atrazine, cyanazine and simazine) in soil and the LODs were in the range of 0.5–1.0 mg/L [8]. Fernandez-Alvarez, et al. used gas chromatography with micro-electron-capture detection (GC-MECD) to detect 36 pesticides in soil and the LODs reached the level of 0.001 mg/kg [9]. Rashid, et al. applied GC–MS methods to determine 19 organochlorine (OC) pesticides and the LODs achieved 0.0003 mg/kg [10]. Overall, although these methods achieved high sensibility, several bottlenecks occur as a result of current techniques such as complicated pretreatment, time-consuming detection, bulky instruments and expensive reagents [11]. Thus, a reliable method is urgently needed for the accurate determination of soil pesticide residue.

Compared with traditional methods, spectroscopy shows great potential in the rapid detection of pesticide residues. Some studies [12,13] have used near infrared reflectance spectroscopy (NIRS) and ultrasensitive fluorescent sensors to detect OC pesticides and the LODs reached 12 mg/kg and 0.0018 mg/L, respectively. Surface enhanced Raman spectroscopy (SERS), as an advanced spectroscopic technique that has emerged over the last decades, has shown great capability on ultrasensitive and label-free chemical or biochemical analysis [14]. SERS is a powerful vibrational spectroscopy technique that allows for highly sensitive structural detection of low concentration analytes through the amplification of electromagnetic fields generated by the excitation of localized surface plasmons [15,16,17]. Currently, metallic nanomaterials, such as silver nanoparticles (AgNPs), gold nanoparticles (AuNPs), and copper nanoparticles (CuNPs), have been widely used as SERS-active colloids [18], whose performance is influenced by the material, size, arrangement, shape of the nanostructures and the excitation wavelength. Among them, AgNPs and AuNPs are the most-widely used SERS colloids to detect pesticides with the mg/kg level LOD due to their superior localized surface plasmon properties [19]. The traditional preparation method of AuNPs is trisodium citrate reduction, also known as the R. Grwith Freema [20] method. According to this method, the diameter of AuNPs are generally in the range of 10–100 nm. However, the morphology, size and physicochemical properties of AuNPs are affected by reaction time, dosage of trisodium citrate and heating temperature. Dong, et al. used the prepared AuNPs (100 mL of 50 mg/L chloroauric acid solution and 4 mL of 5 mg/mL trisodium citrate) as SERS colloids for the detection of DM pesticide in strawberries, the LOD reached 0.1 mg/L [21]. Chen, et al. synthesized AuNPs (100 mL of 2.5 × 10^−4^ M chloroauric acid solution and 5 mL of 1% trisodium citrate) to determine chlorpyrifos pesticide in the apple surface using SERS, the LOD reached 0.0035 μg/cm^2^ [22]. Xu, et al. applied SERS to detect chlorpyrifos pesticide in water based on the prepared AuNPs (25 mL of 2.5 × 10^−4^ M chloroauric acid solution and 1 mL of 0.2 M potassium carbonate), the LOD achieved 0.35 mg/L. Peng et al. achieved the identification and determination of DM in apples using SERS and the LOD of pesticide residues in the apple surface could reach 0.005 μg/cm^2^ [23]. Although the AuNPs mentioned above can basically meet the detection requirements, the traditional fabrication as well as the operation of AuNPs tends to induce signal fluctuations and reproducibility reduction during the SERS detection process. Therefore, in this study, we determined the amount of reagent used to prepare AuNPs, detailed experimental conditions, and explored the influence of heating conditions and time on the aggregation effect of AuNPs.

Here in this study, it is the first time that the SERS technique was used for the determination of DM and CBF in soil. We describe a very simple method for preparing ultrasensitive and reproducible AuNPs by improved reduction of chloroauric acid by the trisodium citrate method, aiming to basically solve the problems of quantitative detection of DM and CBF residues in soil as well as the improvement of SERS determination accuracy. Using the prepared colloids, the SERS signals of DM and CBF were quantitatively analyzed. Overall, it is believed that the prepared AuNPs colloids can become one of the excellent chemical molecules for the ultrasensitive SERS technique, which can provide technical support for the accurate detection of pesticide residues in soil in future studies.

## 2. Results and Discussion

### 2.1. Characterization of Gold Nanoparticles

**Figure 1:**
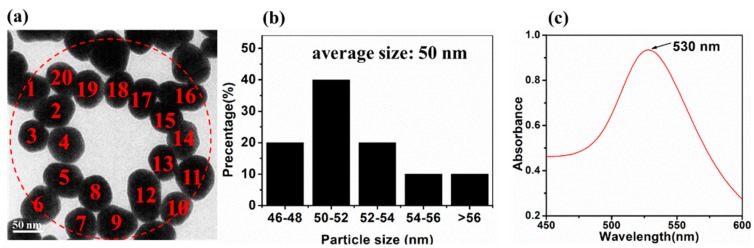
(**a**) The transmission electron microscopy (TEM) images of gold nanoparticles (AuNPs) with heating reaction time at 25 min; (**b**) the distribution of AuNPs; (**c**) the UV–vis spectrometry of AuNPs.

In order to characterize the AuNPs prepared in this study, Figure 1 displays the transmission electron microscopy (TEM) images, the dispersibility and the UV–vis spectrometry of AuNPs, respectively.

It can be clearly seen that the average diameter of as-synthesized AuNPs was about 50 nm in the red-cycle area. The reaction time of the synthesized AuNPs was related to the sufficient degree of both redox reaction and AuNPs agglomeration. As shown in Figures S1 and S2, the AuNPs performed to be aggregated when the heating reaction time was 10 min and 30 min. The dispersibility of the AuNPs reached the optimal status and the diameter of AuNPs was the biggest when the heating reaction time was 25 min. In addition, Figure 1c and Figure S3 show that the appearance of a sharp absorption band was 525–540 nm when the heating reaction time was in the range of 10–25 min within the band of AuNPs ranged from 450–600 nm. And the AuNPs absorbance at the absorption peak ranged from 0.708–0.974, which indicated that it was reliable for fabricating AuNPs by the improved reduction of chloroauric acid according to the trisodium citrate method.

### 2.2. The SERS Performance of the Colloids

**Figure 2:**
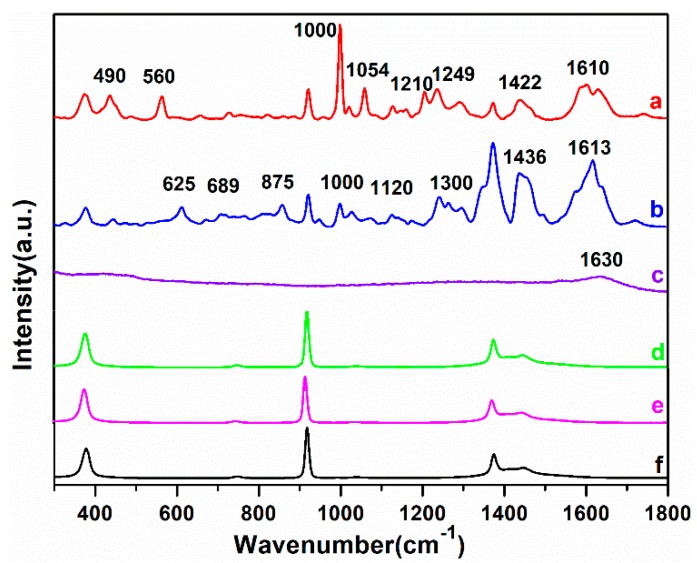
(**a**) The surface enhanced Raman scattering (SERS) spectra of deltamethrin (DM); **(b**) the SERS spectra of carbofuran (CBF); (**c**) the Raman spectra (RS) of AuNPs; (**d**) the RS spectra of DM without AuNPs; (**e**) the RS spectra of CBF without AuNPs; (**f**) the RS spectra of acetonitrile.

In this paper, a 10 mg/L solution of DM and CBF were used to estimate the SERS activity and stability of the AuNPs colloids. Figure 2 shows the SERS spectra of DM and CBF with AuNPs, the Raman spectra (RS) of AuNPs, the RS spectra of DM and CBF without AuNPs and the RS spectra of acetonitrile, respectively.

As shown in Figure 2d–f, except for 378, 922 and 1374 cm^−1^ assigned to acetonitrile which was used to dissolve DM and CBF powder, there were no signals of the RS of DM and CBF. Beside this, the RS of AuNPs only had a faint signal at 1630 cm^−1^ (Figure 2c), indicating that AuNPs had no strong Raman characteristic peaks and did not have a negative effect on experimental results. However, in the case of AuNPs added, the characteristic peaks of DM and CBF were markedly improved. The characteristic peaks of DM molecules located at 490, 560, 1000, 1054, 1210, 1249, 1442 and 1610 cm^−1^ and of CBF molecules located at 625, 689, 875, 1000, 1120, 1300, 1436 and 1613 cm^−1^ were all detected. For confirming the accuracy of SERS spectra, the molecular structure of DM and CBF as well as the Raman spectral simulations were carried out based on density functional theory (DFT) with the assistance of Gaussian.v09 software [24]. Besides, Figure S4 shows the RS of DM and CBF based on DFT calculation and the RS of DM and CBF powder. And the vibrational mode of the main peaks of DM and CBF are shown in Table 1.

According to the DFT calculation, the characterization of Raman characteristic peaks of DM and CBF with their vibrational forms can be acquired. Among the Raman characteristic peaks of DM, the peak at 490 cm^−1^ was assigned to the stretching vibration of benzene ring, the peak at 560 cm^−1^ was assigned to the stretching vibration of C– Br and C–C, the peak at 1000 cm^−1^ was assigned to the stretching vibration of benzene ring and C–C, and the peaks at 1054 and 1210 cm^−1^ were assigned to the stretching vibration of benzene ring and C–C, 1249 and 1442 cm^−1^ were assigned to the stretching vibration of CH_3_, 1613 cm^−1^ was assigned to the stretching vibration of C=C. The results were in good agreement with previous studies [21,25]. Among the Raman characteristic peaks of CBF, the peak at 625 cm^−1^ was assigned to the stretching vibration of benzene ring and C–C–C, the peak at 689 cm^−1^ was assigned to the stretching vibration of benzene ring, the peak at 875 cm^−1^ was assigned to the breathing vibration of N–C=O, the peak at 1000 cm^−1^ was assigned to the breathing vibration of benzene ring and C–C, the peaks at 1120 and 1300 cm^−1^ were assigned to the stretching vibration of CH_3_, the peak at 1436 cm^−1^ was assigned to the stretching vibration of C_4_H_4_O, the peak at 1613 cm^−1^ was assigned to the breathing vibration of benzene ring, which were in good agreement with the Huang’s study [26].

To investigate the sensitivity and stability of the prepared AuNPs colloids for the detection of DM and CBF, the representative SERS spectra of DM and CBF solutions at different concentrations ranging from 0.01–10 mg/L were obtained.

**Figure 3:**
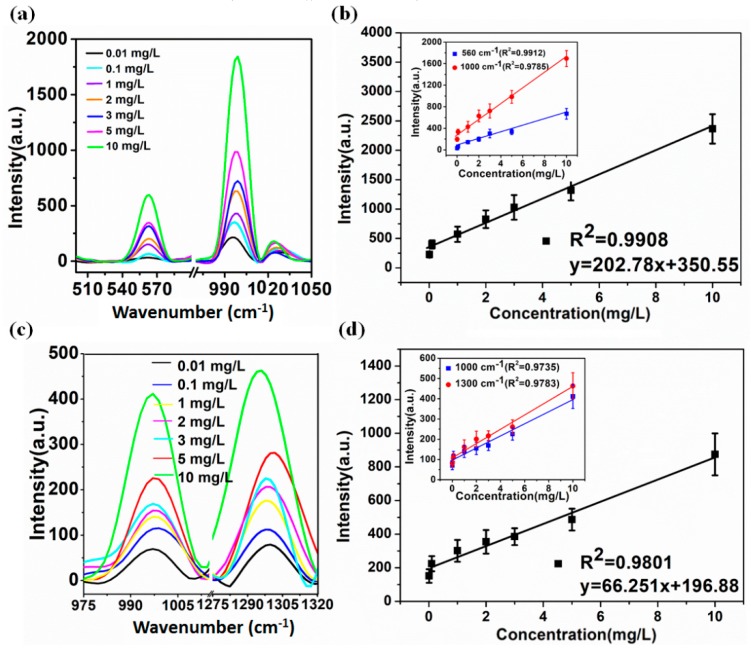
The RS of DM and CBF with its linear equation. (**a**) The RS of DM with AuNPs at different concentrations ranging from 0.01–10 mg/L; (**b**) the linear equation of Raman characteristic peak intensity and its concentration at 560 and 1000 cm^−1^ of the DM molecule; (**c**) the RS of CBF with AuNPs at different concentrations ranging from 0.01–10 mg/L; (**d**) the linear equation of Raman characteristic peak intensity and its concentration at 1000 and 1300 cm^−1^ of the CBF molecule.

As shown in Figure 3a,c, SERS spectra of DM and CBF mixed with the AuNPs were concentration dependent. It was found that the characteristic peaks at 560 and 1000 cm^−1^ of the DM, and 1000 and 1300 cm^−1^ were still identified when the DM and CBF solution concentration was as low as 0.01 mg/L. Figure 3b shows the linear relationship between peak intensity and the concentration of DM at 560 cm^−1^ (*R*^2^ = 0.9912) and 1000 cm^−1^ (*R*^2^ = 0.9785), respectively. The inset in Figure 3b shows the linear relationship between the sum peak intensity of 560 cm^−1^ and 1000 cm^−1^ and the concentration of DM (*R*^2^ = 0.9908). Figure 3d shows the linear relationship between peak intensity and the concentration of CBF at 1000 cm^−1^ (*R*^2^ = 0.9735) and 1300 cm^−1^ (*R*^2^ = 0.9783), respectively. The inset in Figure 3b shows the linear relationship between the sum peak intensity of 1000 cm^−1^ and 1300 cm^−1^ and the concentration of CBF (*R*^2^ = 0.9908). The error bars were obtained from 5 measurements, which proved that the synthetic AuNPs in this study could be served as a good SERS colloid for DM and CBF detection with ultra-sensitivity and reproducibility.

### 2.3. SERS Signal Enhancement Based on the Proportion of AuNPs and DM and CBF Solution

**Figure 4:**
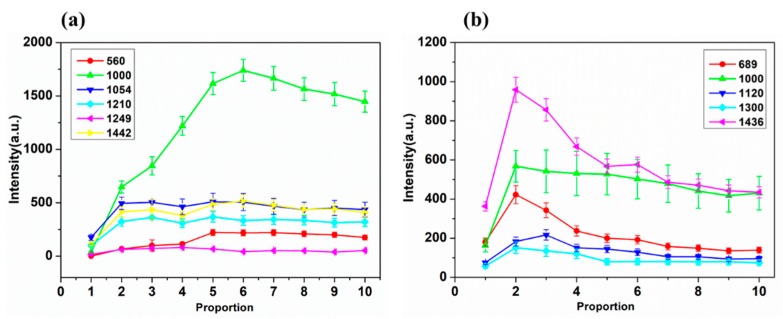
The signal intensity of DM and CBF with AuNPs at different ratios. (**a**) The signal intensity of DM at 560, 1000, 1054, 1210, 1249 and 1422 cm^−1^ with AuNPs and DM at the ratio of 1:1 up to 10:1. (**b**) The signal intensity of CBF at 689, 1000, 1120, 1300 and 1436 cm^−1^ with AuNPs and CBF at the ratio of 1:1 up to 10:1.

It is well known that particle aggregation can greatly enhance the Raman signals of target species located at the conjunction of particles by the so-called “hot spots” effect [27]. The signal intensity of DM and CBF based on different proportion of AuNPs with DM and CBF solution are given in Figure 4.

Figure 4a shows the variation trend of SERS signal intensity with the increase of AuNPs and DM ratio. When the AuNPs and DM ratio was 6:1, the SERS signal intensity was the highest especially at 560 and 1000 cm^−1^. In addition, Figure 4b shows the variation trend of SERS signal intensity with the increase of AuNPs and CBF ratio. The SERS signal intensity achieved the highest at 2:1 ratio. In terms of the molecular weight of DM and CBF, the molecular weight of DM is 505.24 while the molecular weight of CBF is 221.25. The molecular weight of DM is more than twice of CBF. For two pesticide residue solutions with the same concentration and volume, DM requires more amount of AuNPs to adhere while CBF requires less. In this experiment, when the ratio of AuNPs and DM test solution is between 5:1 and 10:1, the SERS signal of DM shows higher intensity; when the ratio of AuNPs and CBF test solution is between 2:1 and 3:1, the SERS signal of CBF shows higher intensity. 

### 2.4. Quantitative Determination of DM and CBF in Soil Using SERS

In this study, to investigate the feasibility of the prepared AuNPs colloids for the detection of DM and CBF in soil, DM and CBF solutions with the concentrations ranging from 10–0 mg/kg were added to the soil and then the DM and CBF residues were extracted from the soil according to QuEChERS (quick, easy, cheap, effective, rugged, safe). After the samples were treated with the national standard method, the final obtained filtered supernatant was divided into two parts for the ultra high performance liquid chromatography (UHPLC) experiment and SERS measurement respectively. The SERS spectra of DM and CBF with 83 different concentration samples were obtained, and the representative SERS spectra are given in Figures S5 and S6. The UHPLC detection results of 83 samples of DM and CBF in soil with different concentrations are shown in Table 2.

**Figure 5:**
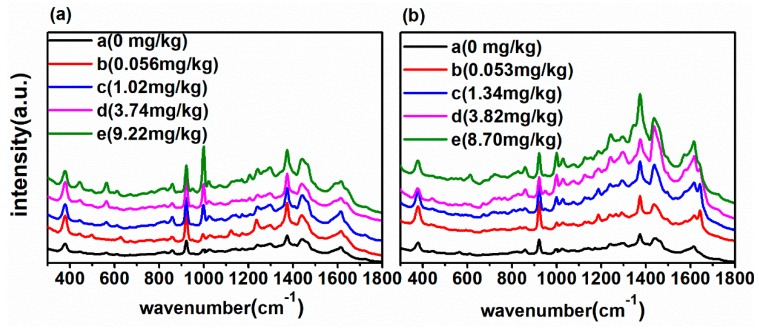
(**a**) The SERS spectra of DM in soil at the concentration of 0, 0.056, 1.02, 3.74 and 9.22 mg/kg; (**b**) the SERS spectra of CBF in soil at the concentration of 0, 0.053, 1.34, 3.82 and 8.70 mg/kg.

According to Table 2, the DM in soil detected by UHPLC was in the range of 0–9.21 mg/kg, and the CBF in soil detected by UHPLC was in the range of 0–8.01 mg/kg. In order to realize the quantitative determination of DM and CBF in soil using SERS as well as investigate the level of LODs, Figure 5a shows the SERS spectra processed with DM in soil at the concentration of 0, 0.056, 1.02, 3.74 and 9.22 mg/kg. Figure 5b shows the SERS spectra of CBF in soil at the concentration of 0, 0.053, 1.34, 3.82 and 8.70 mg/kg.

**Figure 6:**
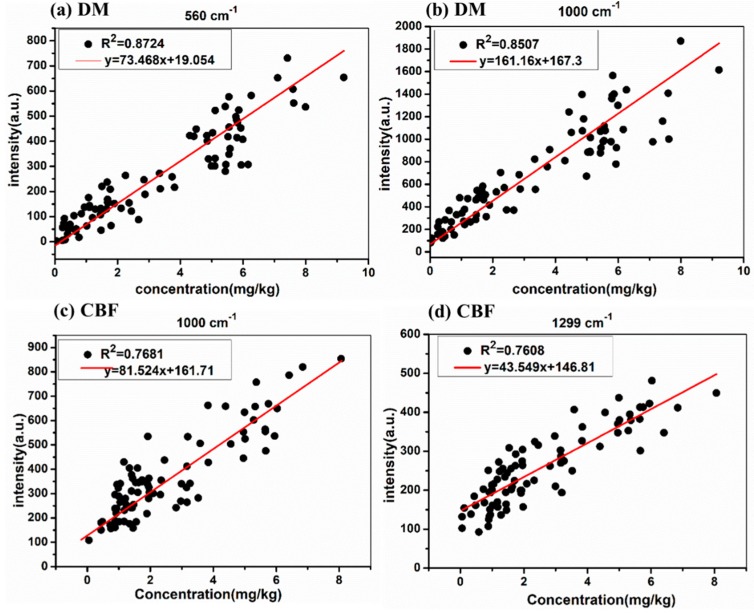
The linear equations of Raman characteristic peaks and its concentration of DM at 560 cm^−1^ (**a**) and 1000 cm^−1^ (**b**); the linear equations of Raman characteristic peaks and its concentration of CBF at 1000 cm^−1^ (**c**) and 1299 cm^−1^ (**d**).

As can be seen in Figure 5, with the decrease of DM concentration in soil from 9.22 to 0 mg/kg at 560 and 1000 cm^−1^ and CBF concentration in soil from 8.70 to 0 mg/kg at 1000 and 1300 cm^−1^, the DM and CBF residues in soil could be detected as low as the concentration of 0.056 mg/kg and 0.053 mg/kg, respectively, which is lower than the maximal residue of pesticides in soil at the level of mg/kg. The Raman peaks in the range 1100–1700 cm^−1^ were independent by the concentration. The reason might be that Raman signals from soil background interfere with this. Furthermore, the linear equations of Raman characteristic peaks and its concentration of the DM and CBF in soil were established. Figure 6a,b present the linear equations of Raman characteristic peaks and its concentration at 560 and 1000 cm^−1^ of the DM in soil, and the linear equations of Raman characteristic peaks and its concentration at 1000 and 1299 cm^−1^ of the CBF in soil are shown in Figure 6c,d.

According to Figure 6a,b, the linear regression equation of Raman characteristic peaks achieved good correlation at 560 and 1000 cm^−1^ peaks (0.8507 < *R*^2^ < 0.8724), which indicates that 560 and 1000 cm^−1^ can be determined as quantitative characteristic peaks of DM in soil. The linear regression equation of Raman characteristic peaks achieved good correlation at 1000 and 1299 cm^−1^ peaks (0.7681 < *R*^2^ < 0.7608) as well, indicating that the Raman characteristic peaks at 1000 and 1299 cm^−1^ can be determined as quantitative characteristic peaks of CBF in soil.

### 2.5. Quantitative Determination of DM and CBF in Soil with PLS

In order to improve the detection accuracy, the partial least squares (PLS) prediction model was established based on SERS spectra. The SERS spectra of DM and CBF of 83 samples at 400–1700 cm^−1^ were obtained and then pretreated with the baseline correction (BC), Savitzky–Golay (S-G), multiplicative scatter correction (MSC), standard normal variation (SNV), and 1st-Derivative (1st-Der), respectively, and then modeled by PLS. The sample set portioning based on the joint x-y distance (SPXY) [28] method was used to separate the soil samples into calibration set and validation set at the ratio of 2:1. The PLS modeling results based on the 400–1700 cm^−1^ spectra under different pretreatments are presented in Table 3.

First, from the perspective of prediction effect of two pesticides, regardless of which pretreatment method and chemometric model were used, the predictive effect of DM model was better (Rp^2^ > 0.90) than that of CBF model (0.78 < Rp^2^ < 0.86). A possible explanation is that the lack of C–Cl, P–O–C and P=S groups in CBF renders the poor affinity of CBF to AuNPs, resulting in the poor quantitative detection effect as well. Second, from the perspective of the modeling results before and after the BC processing, the modeling effect of SERS spectra has been improved after the BC processing as well as S-G smoothing, MSC, SNV and 1st-Der. The reason for this could be that the baseline drift with different concentrations of pesticide residues in soil is serious, the high baseline of some pesticides in low concentration might misjudge the modeling effect, suggests that the baseline processing of SERS spectra is essential for obtaining the accurate modeling results. Third, from the perspective of pretreatment methods, the SERS spectra with different pretreatment methods shows different modeling effect, and the modeling effect of the same pesticide is similar after using different pretreatment methods. Among them, the SERS original spectra perform a slightly better modeling effect after MSC and SNV treatment compared with S-G smoothing and 1st-Der. The reason could be that MSC and SNV eliminated the effects of uneven sample distribution and filling density, which improved the spectral resolution, reduced standard deviation between samples and separated the main characteristic peaks for quantitative analysis. In general, the PLS model of SERS spectra of deltamethrin in soil processed with BC and MSC achieved the best prediction effect (Rp^2^ = 0.9176, RMSEP = 0.604, RPD = 3.3741), and the PLS model of SERS spectra of CBF in soil obtained the best prediction effect (Rp^2^ = 0.8517, RMSEP = 0.560, RPD = 2.3827).

### 2.6. Model Accuracy Verification 

To verify the accuracy of the determination method of soil pesticide residue performed in this study, four soil extract samples with unknown DM and CBF pesticides concentration were pretreated with Raman characteristic peaks (DM: 560 cm^−1^; CBF: 1000 cm^−1^) and PLS model, respectively. Table 4 presents the results between the real value and predicted value of DM and CBF pesticides in soil.

According to Table 4, the recovery of DM were 76.0% to 106.0%, 84.0% to 106.0%, and 85.0% to 97.86%, respectively. The recovery of CBF were 82.50% to 102.6%, 80.0% to 87.63%, and 86.0% to 97.75%, respectively. It is further demonstrated that the application of the SERS technique for determining soil pesticide residue is reliable and effective, which shows great potential in pesticide residue detection of soil.

## 3. Materials and Methods 

### 3.1. Chemicals

In this experiment, the chemical reagents include DM (99.6% purity, Sigma-Aldrich, Beijing, China), CBF (99.8% purity, Sigma-Aldrich, Beijing, China), acetonitrile (chromatographically pure, Amethyst Chemicals, Beijing, China), trisodium citrate, chloroauric acid (ethylenediamine-N-propylsilane), anhydrous sodium acetate, C18 and graphite carbon black (Analytical Purity, Chemical Reagent Beijing Co., Ltd., Beijing, China). 

### 3.2. Instrumentation

The SERS spectra were obtained by an RmTracer-200-HS portable Raman spectrometer combined with a 785 nm excitation wavelength diode-stabilized stimulator (Opto Trace Technologies, Inc., Mountain View, CA, USA). The optical absorption measurements were carried out by a TU-1901 Ultraviolet Spectrophotometer (Beijing General Instrument Co., Ltd., Beijing, China). Morphological features of the as-produced AuNPs structures were characterized with an FEI Tecnai G2 F20 S-TWIN transmission electron microscope (FEI Company, Hillsboro, OR, USA). DM and CBF residues in soil were detected using Agilent 1290 Ultra High Performance Liquid Chromatography combined with a Photodiode Array Detector (Agilent Technology Co., Ltd., Santa Clara, CA, USA).

### 3.3. Experimental Methods

#### 3.3.1. Preparation of Gold Nanoparticles

In this study, traditional reduction of high-chloroauric acid by the trisodium citrate method was performed to prepare AuNPs, and the effects of heating reaction time and amount of trisodium citrate on the AuNPs were investigated. The synthesis of AuNPs at different heating reaction times was as follows. First, a 100 mL of 0.01% HAuCl_4_ solution was heated for boiling on a constant magnetic stirrer, and then 0.5 mL of 1% of Na_3_C_6_H_5_O_7_ solution was added. Second, the mixture was stirred at 100 r/min for 10 min, 15 min, 20 min, 25 min and 30 min at boiling status, respectively. Third, when the solution cooled, five gold gel solution was poured into a centrifuge tube and was stored in dark at 4 °C after repeating purification. The nanoparticles prepared in this experiment were stable, and the reinforcing effect of the prepared gold colloid could be maintained about 1 to 2 weeks.

#### 3.3.2. Preparation of Soil Samples

The acidic red soil samples were collected from Qingyuan county, Zhejiang province, China (N 34°44′, E 127°45′), which were air-dried and sieved by a 60-mesh (0.028 mm) sifter originally. Then the soil samples were mixed with DM and CBF in different concentrations (0–10 mg/kg) and air-dried again. Referred to QuEChERS [29], the method used to extract DM and CBF residues from soil was as follows. First, 5 mL ultra-pure water was mixed with 10 g soil sample, and the mixture was placed in a 50 mL centrifuge tube for 30 s vortex oscillation. Second, 10 mL of 1% acetonitrile was added and vortexed for 3 min at 400 r/min, and then performed with 2 min ultrasonic oscillation. Third, 3 g sodium chloride and 4 g sodium acetate were added orderly after 15 min standing, then the solution was vortexed for 1 min at 400 r/min and centrifuged for 5 min at 5000 r/min. Fourth, 1.5 mL supernatant, 150 mg magnesium sulfate, 50 mg PSA, 10 mg graphite carbon black and 50 mg C_18_ were added in turn. Then the supernatant was centrifuged for 1 min to remove proteins, fats, carbohydrates and other interferential substances. Finally, the solution was centrifuged for 5 min at 5000 r/min and the supernatant was filtered through a 0.22 μm organic membrane. The obtained supernatant was used for Ultra High Performance Liquid Chromatography (UHPLC) and SERS measurement.

#### 3.3.3. UHPLC Measurement

For validation of the SERS method, an Agilent 6410 UHPLC instrument (Agilent Technology Co., Ltd., Santa Clara, CA, USA) equipped with a quaternary solvent delivery system, an autosampler, a column thermostat, a degasser unit and a diode array detector (DAD) were used to measure DM and CBF samples prepared the same as Raman samples preparation in Section 3.3.2. The analytical column (Agilent ZORBAX SB-C18, 150 mm × 2.1 mm × 3.5 μm) was maintained at 30 °C and eluted isocratically with a mixture of methanol and water (50:50 *v*/*v*) at a total flow rate of 0.3 mL/min. The elution was monitored at 300 nm.

#### 3.3.4. SERS Measurement

The RS of DM and CBF in powder as well as the SERS measurements were carried out using an RmTracer-200-HS portable Raman spectrometer system combined with a 785 nm excitation wavelength diode-stabilized stimulator. Before RS acquisition, the instrument should be calibrated using a 785 nm excitation wavelength with acetonitrile at 374, 922, 1374 and 2252 cm^−1^. The parameters were set as follows: A power of 200 mw, a scanning range of 200–3300 cm^−1^, an optical resolution of 2 cm^−1^, an integration time of 5 s and an average spectral value of 3 times. The RS collection of solid DM and CBF were that DM and CBF powder were in quartz plate with glass slides flattened and the spectra were acquired with matching microscope platform. According to the Section 2.3, when collecting the SERS of DM samples, 600 μL gold colloid, 100 μL test solution and 100 μL potassium chloride were added in turn into a 2 mL quartz bottle, then it was placed at a liquid sample pool. When collecting the SERS of CBF samples, 200 μL gold colloid, 100 μL test solution and 100 μL potassium chloride were added in turn into a 2 mL quartz bottle, then it was placed at a liquid sample pool. Potassium bromide can prevent the excessive agglomeration of gold nanoparticles and improve the stability and repeatability of SERS signal enhancement [30].

### 3.4. Spectral Preprocessing Methods

The SERS spectra were susceptible to fluorescence background, thus removing background fluorescence from Raman signals is essential for analyzing Raman signals accurately. Currently, to investigate the SERS determination of pesticide residues based on AuNPs, each original SERS spectrum was processed with S-G [31] 5 points smoothing filter and then conducted with BC. Therefore, for the better determination of the SERS spectra of DM and CBF residues in soil quantitatively, here in the PLS model, each original SERS spectrum was processed by the S-G 5 points smoothing filter and BC, and then it was pretreated with the MSC, SNV, and 1st-Der, respectively. The principle of the SNV [32] algorithm is that the absorbance values of each wavelength point satisfies a certain distribution in each spectra, and the spectral correction was performed according to this assumption. The basic idea of the MSC [33] algorithm is to use an ideal spectra to represent all the samples, and the original spectra is corrected with the slope and intercept of the linear equation.

### 3.5. Partial Least Squares Model

PLS is a most commonly-used calibration model for spectral data analysis, which reflects the relationship between spectra and attribution information due to its flexibility and reliability [34,35]. When PLS is applied to dealing with spectral data, the spectral matrix is decomposed first and the main principal component variables are obtained, then the contribution of each principal component is calculated. The flexibility of PLS makes it able to interpret the dependent and independent variables well by establishing regression models. In this study, the PLS model was established with the spectral data as *X* and the measured content of DM and CBF by UHPLC as *Y*, whose best principal factor was determined by the root mean square error of cross validation (RMSECV).

### 3.6. Model Evaluation Index

In this experiment, the modeling effect was evaluated by the coefficient of determination (R^2^), the root mean square error (RMSE) and the residual predictive deviation (RPD). The coefficient of determination *R*^2^ reflects the level of intimacy between variables, the RMSE reflects the model accuracy, and RPD reflects the predictive ability of the model. The higher the RPD, the lower the RMSE, and the closer the *R*^2^ is to 1, the better the performance of the prediction model. In this study, R_C_^2^ and R_P_^2^ represent the coefficient of the determination of the calibration set and the prediction set respectively, while RMSEC and RMSEP represent the root mean square error of the calibration set and the prediction set respectively. Besides, the RPD was suggested to be at least 3 for agriculture applications; while RPD between 2 and 3 indicated a model with good predictive ability; RPD between 1.4 and 4 indicated an intermediate model which requires improvement; and RPD lower than 1.4 indicated the poor predictive ability [36]. In addition, all above-mentioned data analysis in this study were performed on OMNIC v8.2 (Thermo Nicolet Corp., Madison, WI, USA), MATLAB R2014a (The MathWorks, Inc., Natick, MA, USA) and Gaussian.v09 (Gaussian, Inc., Wallingford, CT, USA).

## 4. Conclusions

In this paper, we described a very simple method for the preparation of the suitable AuNPs colloids and applied the SERS for the quantitative determination of DM and CBF in soil initially. DM and CBF residues in soil were quantitatively predicted based on certain specific peaks and the LODs reached 0.056 mg/kg and 0.053 mg/kg, respectively. And the prediction of the PLS model was better than that of the single variable model. Here in this study, we demonstrated the promising potential of the SERS method to effectively detect pesticides in soil and reached the detection limit of soil pesticide residues. However, there is still room for improvement in terms of the sensitivity of SERS for the improvement of LOD to the μg/kg level. Therefore, further studies could focus on the enhancement of LOD, as well as the preparation approach to obtain higher SERS enhancements.

## Figures and Tables

**Table 1:** Characterization of Raman characteristic peaks of DM and CBF with their vibrational forms.

Deltamethrin				Carbofuran			
SERS (cm^−1^)	DFT (cm^−1^)	Powder (cm^−1^)	Assignment	SERS (cm^−1^)	DFT (cm^−1^)	Powder (cm^−1^)	Assignment
490	500	495(w)	υ_ring_	625	633	631(w)	υ_ring_+υ(C–C–C)
560	553	554(m)	υ(C–Br) + υ(C–C)	689	711	690(m)	υ_ring_
1000	987	1000(vs)	υ_ring+_ δ(C–C)	875	872	876(m)	υ(N–C=O)
1054	1065	1045(w)	υ(C–C)	1000	1005	1009(m)	υ_ring+_ δ(C–C)
1210	1191	1206(m)	υ(C–C)	1120	1112	1110(m)	δ(CH_3_)
1249	1248	1250(w)	υ_ring_+δ(C–H)	1300	1304	1295(m)	δ(CH_3_)
1442	1440	1446(w)	δ(CH_3_)	1436	1473	1446(s)	υ(C_4_H_4_O)
1610	1613	1610(m)	υ(C=C)	1613	1625	1603(s)	υ_breathe_

Note: vs. = very strong; s = strong; m = medium; w = weak; υ = stretching; δ = deformable vibration.

**Table 2:** The ultra high performance liquid chromatography (UHPLC) detection results of 83 samples of DM and CBF in soil with different concentrations.

Samples	Added (mg/L)	UHPLC (mg/kg)	UHPLC RSD (%)	UHPLC Recovery (%)
DM	0–10	0–9.21	0.36–20.3	69.8–98.36
CBF	0–10	0–8.01	0.51–23.1	70.2–90.52

**Table 3:** PLS modeling results based on the full spectra under different pretreatments.

Sorts	Baseline Correction	Pretreatment	Calibration		Prediction		
			Rc^2^	RMSEC	Rp^2^	RMSEP	RPD
DM	Before	SG	0.9031	0.7229	0.8800	0.856	2.8239
		MSC	0.9027	0.6992	0.8540	0.944	2.6108
		SNV	0.8942	0.7774	0.8932	0.875	2.6049
		1^st^-Der	0.8892	0.8205	0.9124	0.690	3.1761
	After	SG	0.9752	0.3821	0.9059	0.673	3.0182
		MSC	0.9841	0.3002	0.9176	0.604	3.1741
		SNV	0.9769	0.3640	0.9099	0.716	3.0944
		1^st^-Der	0.9686	0.4231	0.9151	0.677	3.2258
CBF	Before	SG	0.7515	0.9752	0.6593	1.160	1.7411
		MSC	0.7517	0.9685	0.6768	1.170	1.7307
		SNV	0.7441	0.9498	0.6757	1.210	1.7584
		1^st^-Der	0.6869	1.0200	0.7868	1.080	2.0414
	After	SG	0.8558	0.9740	0.8350	0.568	2.3299
		MSC	0.9016	0.7568	0.8517	0.560	2.3827
		SNV	0.8516	0.9626	0.8212	0.602	2.0977
		1^st^-Der	0.8904	0.8025	0.7843	0.900	1.8150

**Table 4:** Respective determination of DM and CBF pesticides in soil by UHPLC and SERS.

Samples	Added (mg/L)	Peaks (mg/L)	Peaks Recovery (%)	PLS (mg/L)	PLS Recovery (%)	UHPLC (mg/L)	UHPLC Recovery (%)
DM	0.5	0.38	76.0	0.51	106.0	0.48	96.0
	2	2.12	106.0	2.30	115.0	1.70	85.0
	4	3.30	82.5	3.36	84.0	3.81	95.3
	8	6.81	85.2	6.74	84.3	7.59	97.9
CBF	0.5	0.42	84.0	0.4	80.0	0.43	86.0
	2	1.65	82.5	1.75	87.5	1.86	93.0
	4	3.33	83.3	3.35	83.8	3.91	97.8
	8	8.21	102.6	7.01	87.6	7.48	93.5

## Supplementary Materials

Supplementary materials can be found at https://www.mdpi.com/1422-0067/20/7/1731/s1.
