# Supplementary material for: Gold Nanoparticles for Qualitative Detection of Deltamethrin and Carbofuran Residues in Soil by Surface Enhanced Raman Scattering (SERS)
Source: Int J Mol Sci. 2019 Apr 8;20(7):1731. doi: 10.3390/ijms20071731 (PMC6479568; doi:10.3390/ijms20071731)
Supplement: Supplementary file 1 [file ijms-20-01731-s001.pdf]

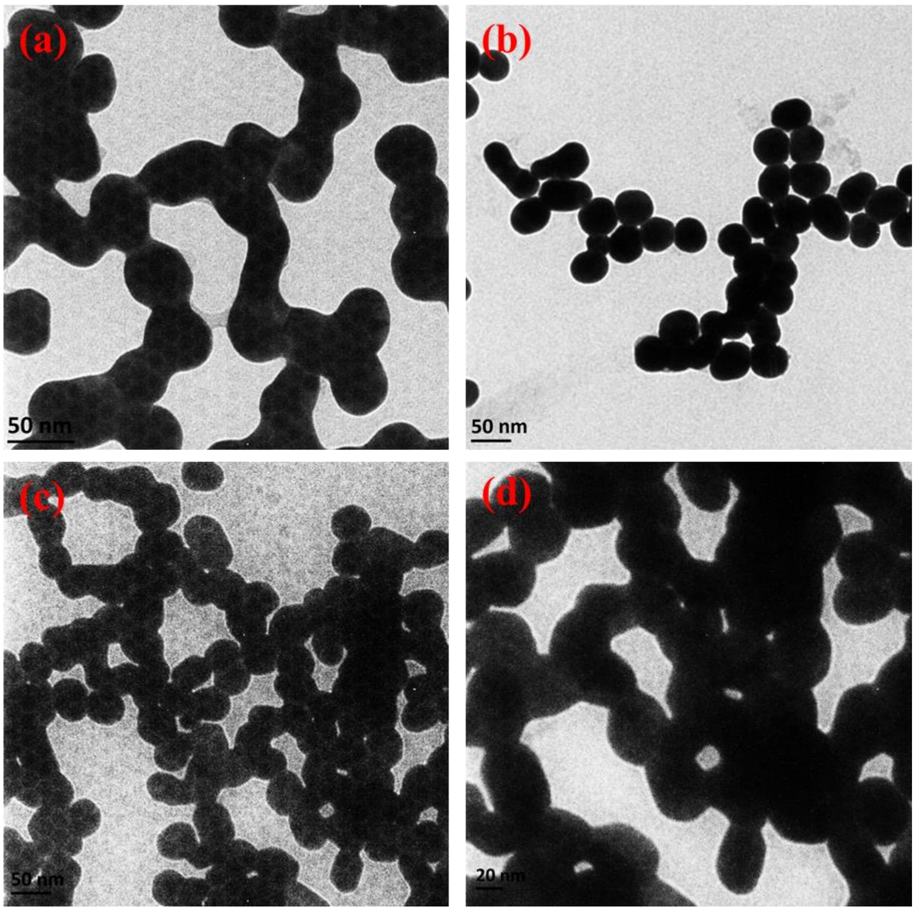

Figure S1. The transmission electron microscopy (TEM) images of AuNPs with different heating reaction time: (a) 10 min; (b) 15 min; (c) 20 min; (d) 30 min.

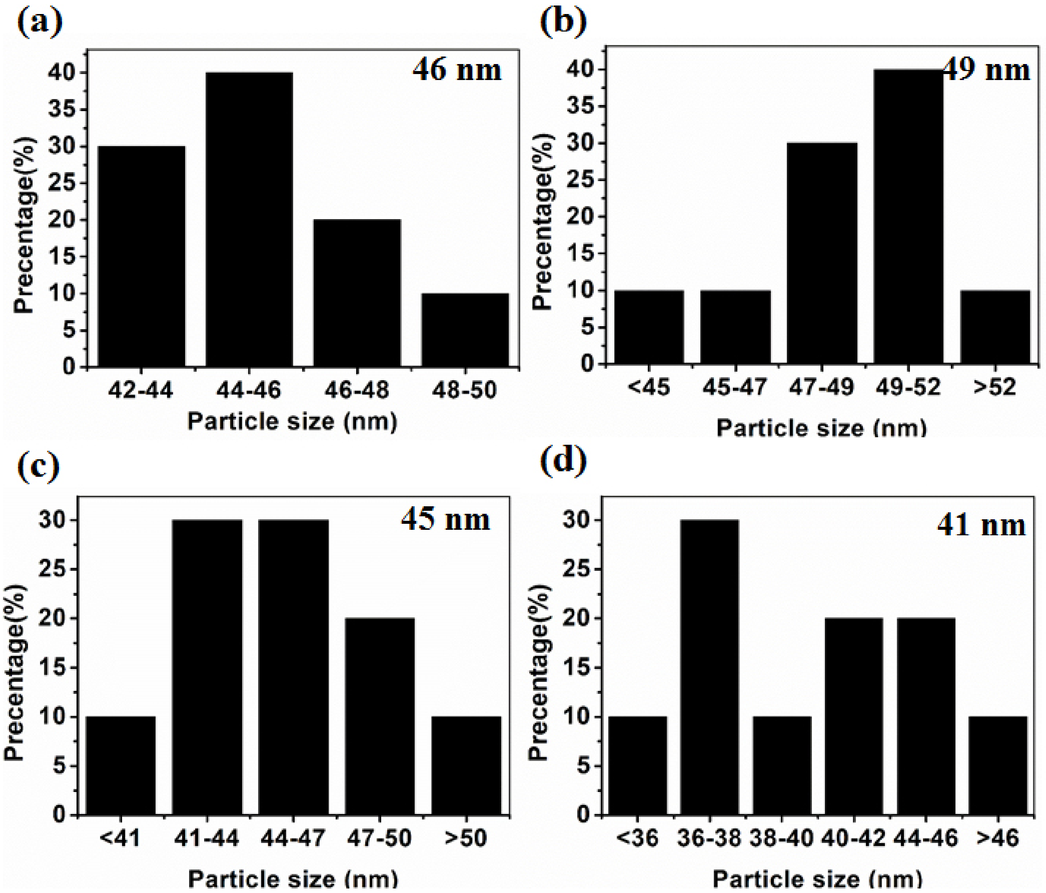

Figure S2. The dispersibility of AuNPs with different heating reaction time: (a) 10 min; (b) 15 min; (c) 20 min; (d) 30 min.

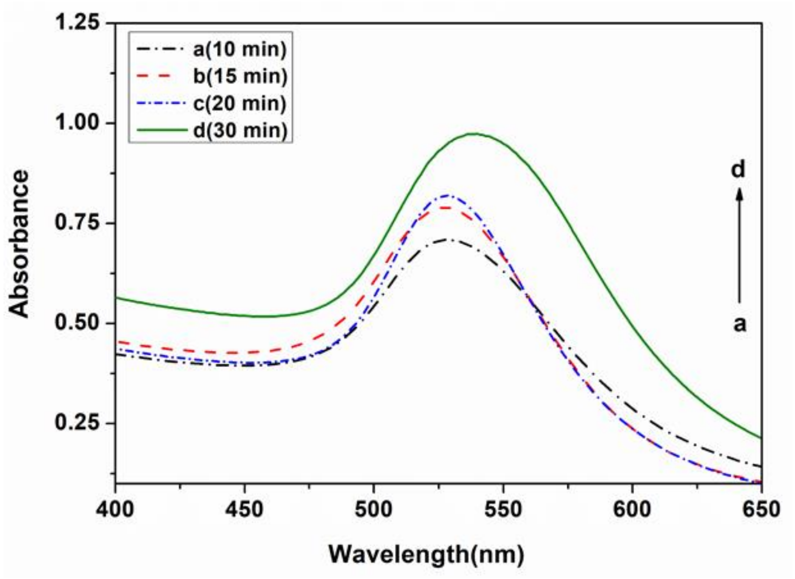

Figure S3. the UV–vis spectrometry of AuNPs with different heating reaction time: (a) 10 min; (b) 15 min; (c) 20 min; (d) 30 min.

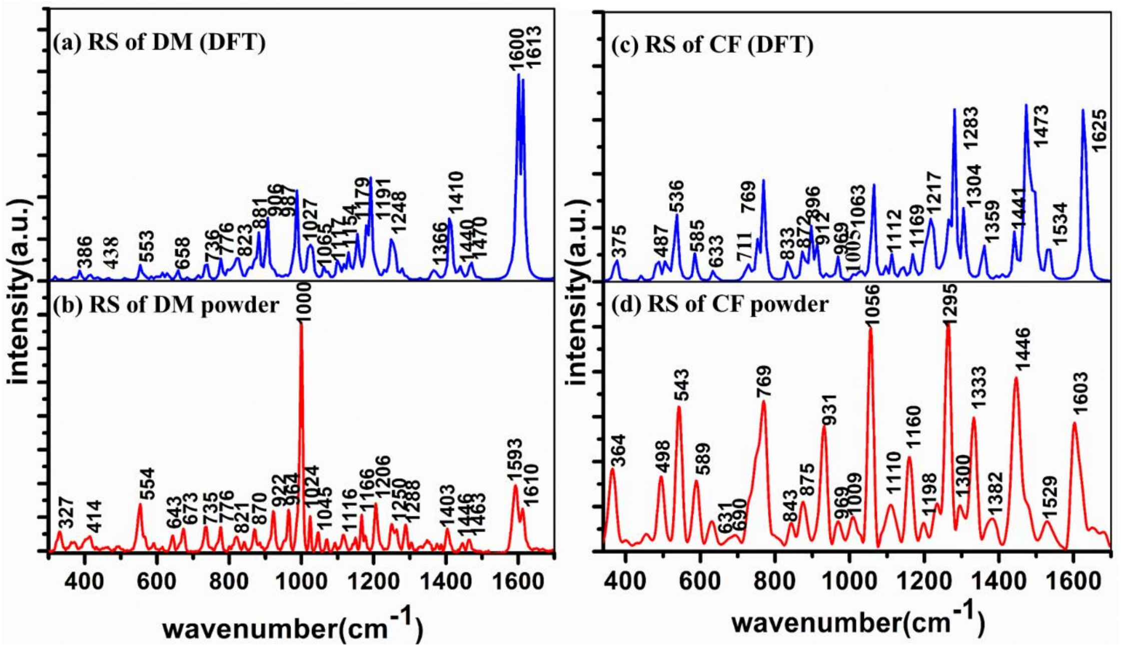

Figure S4. (a) The RS simulation based on DFT calculation; (b) the RS of DM powder; (c) the RS simulation based on DFT calculation; (d) the RS of CBF powder.

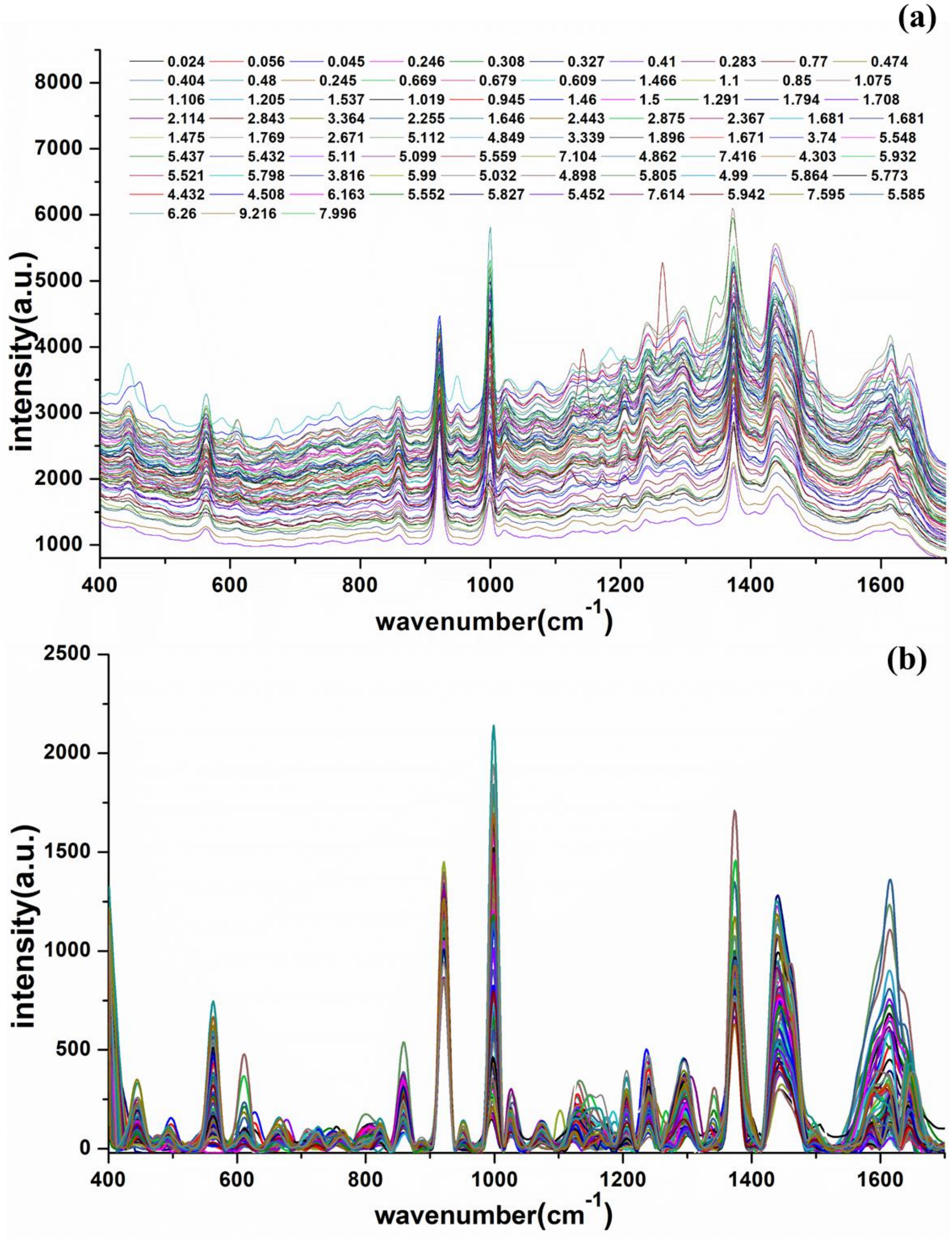

Figure S5. The RS of DM residues in soil: (a) the original SERS spectra of 83 samples; (b) the SERS spectra of 83 samples after baseline correction.

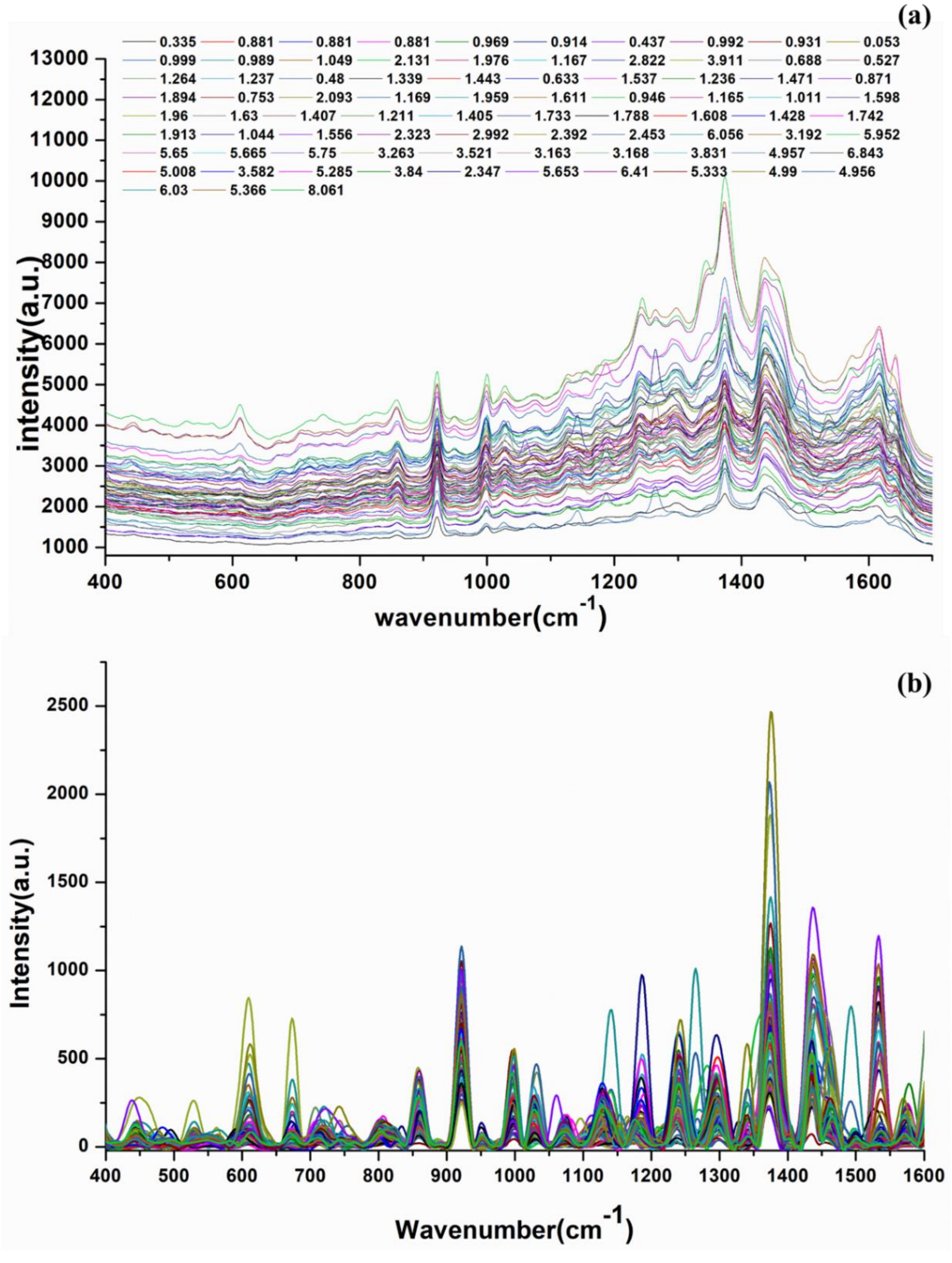

Figure S6. The RS of CBF residues in soil: (a) the original SERS spectra of 83 samples; (b) the SERS spectra of 83 samples after baseline correction.
